# How to make study documents clear and relevant: the impact of patient involvement

**DOI:** 10.1192/bjo.2021.1040

**Published:** 2021-11-04

**Authors:** Sagar Jilka, Georgie Hudson, Sonja M. Jansli, Esther Negbenose, Emma Wilson, Clarissa M. Odoi, Magano Mutepua, Til Wykes

**Affiliations:** Institute of Psychiatry, Psychology & Neuroscience, King's College London, UK; and Division of Mental Health & Wellbeing, Warwick Medical School, University of Warwick, Coventry, UK; Institute of Psychiatry, Psychology & Neuroscience, King's College London, UK; Institute of Psychiatry, Psychology & Neuroscience, King's College London, UK; Institute of Psychiatry, Psychology & Neuroscience, King's College London, UK; and Department of Psychology, South London and Maudsley NHS Foundation Trust, UK; Institute of Psychiatry, Psychology & Neuroscience, King's College London, UK; and Department of Psychology, South London and Maudsley NHS Foundation Trust, UK; Institute of Psychiatry, Psychology & Neuroscience, King's College London, UK; and Department of Psychology, South London and Maudsley NHS Foundation Trust, UK; Institute of Psychiatry, Psychology & Neuroscience, King's College London, UK; and Department of Psychology, South London and Maudsley NHS Foundation Trust, UK; Institute of Psychiatry, Psychology & Neuroscience, King's College London, UK; and Department of Psychology, South London and Maudsley NHS Foundation Trust, UK

**Keywords:** Patient and public involvement, readability, information sheets, jargon, accessibility

## Abstract

**Background:**

Patient and public involvement can improve study outcomes, but little data have been collected on why this might be. We investigated the impact of the Feasibility and Support to Timely Recruitment for Research (FAST-R) service, made up of trained patients and carers who review research documents at the beginning of the research pipeline.

**Aims:**

To investigate the impact of the FAST-R service, and to provide researchers with guidelines to improve study documents.

**Method:**

A mixed-methods design assessing changes and suggestions in documents submitted to the FAST-R service from 2011 to 2020. Quantitative measures were readability, word count, jargon words before and after review, the effects over time and if changes were implemented. We also asked eight reviewers to blindly select a pre- or post-review participant information sheet as their preferred version. Reviewers’ comments were analysed qualitatively via thematic analysis.

**Results:**

After review, documents were longer and contained less jargon, but did not improve readability. Jargon and the number of suggested changes increased over time. Participant information sheets had the most suggested changes. Reviewers wanted clarity, better presentation and felt that documents lacked key information such as remuneration, risks involved and data management. Six out of eight reviewers preferred the post-review participant information sheet. FAST-R reviewers provided jargon words and phrases with alternatives for researchers to use.

**Conclusions:**

Longer documents are acceptable if they are clear, with jargon explained or substituted. The highlighted barriers to true informed consent are not decreasing, although this study has suggestions for improving research document accessibility.

It is important for researchers to provide clear participant information sheets to have true informed consent,^1^ and one way to achieve this is through patient and public involvement. Patient and public involvement is required for UK publicly funded health research, with researchers describing their involvement strategy.^2,3^ Patient and public involvement is defined as ‘research carried out with or by members of the public rather than to, about or for them’,^4^ where the public, patients and carers are active partners in research.^5^ Traditional research models often confine patients to the end of the research ‘pipeline’, but ensuring collaboration across all stages can improve study outcomes, identify appropriate research questions and reduce ‘research waste’.^6–9^ The Feasibility and Support to Timely Recruitment for Research (FAST-R; https://www.maudsleybrc.nihr.ac.uk/patients-public/support-for-researchers/) service offers access to trained mental health patients and carers for improving participant facing documents. This service was set up by the Mental Health Research Network in London in 2011, and is now organised and funded by the National Institute for Health Research (NIHR) Maudsley Biomedical Research Centre. FAST-R is used in the early stages of the research cycle, before seeking funds or ethical approval. The service can speed up these processes by highlighting potential stumbling blocks; for example, by ensuring clear and accessible language in participant-facing documents and including information needed for genuinely informed consent.^10^ Documents are considered by a group of reviewers with a facilitator, and are returned within 7 working days. Patient involvement has been contested despite the supports for co-production,^11,12^ so it is essential to understand whether there is an impact. Several potential measures are unexplored that affect whether someone has informed consent, such as readability and jargon. The US Food and Drug Administration (FDA) recommend a readability grade of eight or lower (i.e. readable by someone aged 13 years).^13^ These quantitative measures do not tell the whole story, as valuable comments on the ways patient and public involvement benefits a study can be missed.^14,15^ Therefore, in addition to quantitative measures, we explored FAST-R members’ views in detail, using qualitative methods.

## Method

### Design

**Fig. 1:**
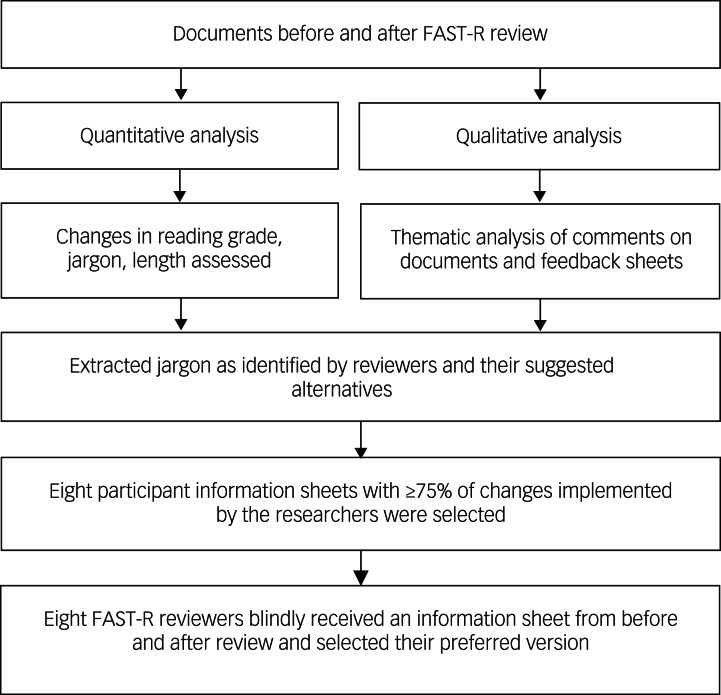
The stages of the study's methodology, including the qualitative and quantitative analyses. FAST-R, Feasibility and Support to Timely Recruitment for Research.

This is a mixed-methods study auditing documents submitted to FAST-R before and after review, and investigating their accessibility (reading grade, jargon, length, etc.) by using quantitative analyses. All study documents were related to mental health research and consisted of a variety of study types and fields, such as clinical trials, cross-sectional investigations and qualitative studies (e.g. Martland et al,^16^ Greer at al^17^). This was supplemented by a detailed qualitative analysis of patient/carer FAST-R member reviews found on the feedback forms and on the documents themselves. The authors selected the participant information sheets with at least 75% of changes implemented by the researcher. These eight studies were reviewed by eight FAST-R reviewers who each considered one study they had not seen before, with no information on which document was pre- or post-review. Each reviewer gave their preferred document and provided reasons for their choice. We extracted jargon words and phrases identified by the reviewers from the pre-review documents as a guide to how changes might be made in the future. This is a secondary data analysis, so no ethical approval was required because no participants were recruited and no reviewers were identifiable. The research was conducted in accordance with the Helsinki Declaration of 1975, as revised in 2008. See Fig. 1 for a visual representation of the study's methodology.

### FAST-R members

Members of the FAST-R service are recruited through a variety of sources, such as local mental health charities, referrals and The Maudsley Biomedical Research Centre's Consent for Contact programme.^18^ All members are interviewed prior to joining the group and given information about the group and their role. Inclusion criteria for membership consists of lived experience of mental health service use and/or being a carer of a mental health patient, as well as some prior experience taking part in research studies. Members are paid and group demographics range in age, ethnicity and socioeconomic status; however, the majority of the reviewers are White British, middle-aged and university-educated.

### Part one: quantitative assessment

#### Procedure

Data consisted of original and final versions of study documents submitted between July 2011 and July 2020.

#### Measures

We used the Flesch–Kincaid Grade Level measure,^19^ which has been used extensively to assess healthcare information.^1,20–22^ The readability score was extracted from Microsoft Word (version 2109 for Windows) before and after review. Higher scores represent more complicated text. Word count and the number of jargon words were calculated where words and phrases were classed as ‘jargon’ if reviewers said they were jargon, needed explaining or simplifying. We calculated changes by subtracting post from pre FAST-R scores so a negative reading grade means they became easier. We also counted the number of suggested changes and the number actually implemented in the final document.

We categorised the documents by type (e.g. participant information sheet, consent form) and investigated whether there were any changes over time.

#### Analysis

We characterised the documents using descriptive statistics. Readability scores, word count and jargon were subjected to paired samples *t*-tests to measure changes after FAST-R review. We investigated the relationship between reading grade, word count, jargon, the number of suggested changes and the percentage of changes implemented, with the year documents were submitted for review, using Pearson correlations to detect changes over time. We also report the differences between document types requiring and implementing changes. All analyses were carried out with SPSS version 26 for Windows.

### Part two: qualitative assessment

#### Procedure

Tracked comments made on the original research documents, as well as comments for researchers on the FAST-R feedback forms, were the basis of all analyses. We selected participant information sheets with at least 75% of changes implemented by the researchers from our total sample. This consisted of eight studies, which we gave to eight FAST-R reviewers who reviewed one study each and selected their preference with reasons for their choice.

#### Analysis

Documents were analysed thematically,^23^ and themes were inductively extracted by two researchers independently, using Pope et al's analysis framework.^24^ This is a five-stage process and involves: stage 1 involves familiarisation with raw data; stage 2 involves identifying a thematic framework; stage 3 involves indexing by applying the thematic framework to all the data by annotating the transcripts; stage 4 involves charting by rearranging the data according to the thematic framework; and stage 5 involves mapping and interpretation by defining concepts, mapping the range and nature of phenomena, and creating typologies.

Stages 1 and 2 were undertaken as a group through discussion to create a coding framework (‘framework category’). To minimise bias and maximise our inductive approach from an emic perspective,^25^ two patient researchers independently undertook inductive coding,^26^ and categorised the codes into emerging themes and subthemes. Mapping and interpretation were repeated with a third researcher, a FAST-R facilitator, who reviewed the coding framework and made the final judgement on emerging themes and subthemes. Analysis was carried out with NVivo version 12 for Windows (QSR International, Melbourne, Australia; see https://www.qsrinternational.com/nvivo-qualitative-data-analysis-software/home).

For reviewers’ blind ratings, we report how many reviewers preferred the post-review participant information sheet, and summarise their feedback on the information sheets.

## Results

### Are there measurable differences in the documents after FAST-R review?

Over the 9 years of data collection, 99 documents had both the pre- and post-review. The most frequent were participant information sheets (*n* = 49) and consent forms (*n* = 29), but there was a range of document types (see Supplementary Table 1 available at https://doi.org/10.1192/bjo.2021.1040).

### Accessibility

Table 1 summarises the outcomes before and after the FAST-R review.

**Table 1:** Reading grade, word count and jargon for all documents before and after FAST-R review

	Before FAST-R mean (±s.d.)	After FAST-R mean (±s.d.)	Mean change (±s.d.)
Reading grade			
All documents (*n* = 99)	10.17 (±2.20)	10.27 (±2.28)	0.11 (±1.53)
Participant information sheet (*n* = 49)	10.08 (±1.04)	10.17 (±1.36)	0.09 (±1.40)
Consent form (*n* = 29)	9.98 (±1.93)	10.48 (±2.05)	0.50 (±1.47)
Lay summary (*n* = 9)	13.39 (±2.82)	12.67 (±3.82)	−0.72 (±2.30)
Topic guide (*n* = 7)	7.73 (±2.88)	8.2 (±2.91)	0.47 (±0.68)
Questionnaire (*n* = 2)	5.40 (±0.71)	5.3 (±0.71)	−0.10 (0)
Protocol (*n* = 1)	14.3	10.3	−4
Assent form (*n* = 1)	12.10	11.1	−1
Invitation letter (*n* = 1)	11.8	11.5	−3
Word count			
All documents (*n* = 99)	1199.42 (±1116.83)	1353.32 (±1336.36)	153.90 (±329.88)
Participant information sheet (*n* = 49)	1895.82 (±1114.89)	2171.22 (±1385.99)	275.41 (±417.92)
Consent form (*n* = 29)	309.41 (±151.54)	347.76 (±172.28)	38.34 (±99.30)
Lay summary (*n* = 9)	625.67 (±376.09)	590.78 (±341.28)	−34.89 (±130.37)
Topic guide (*n* = 7)	801.29 (±614.95)	920 (±829.42)	118.71 (±236.57)
Questionnaire (*n* = 2)	792 (±832.97)	797.50 (±846.41)	5.5 (±13.44)
Protocol (*n* = 1)	3443	3532	89
Assent form (*n* = 1)	395	378	−17
Invitation letter (*n* = 1)	213	242	−29
Jargon			
All documents (*n* = 99)	1.68 (±8.24)	0.98 (±5.44)	−0.70 (±2.92)
Participant information sheet (*n* = 49)	2.14 (±11.26)	1.18 (±7.29)	−0.96 (±4.00)
Consent form (*n* = 29)	0.86 (±3.90)	0.69 (±3.34)	−0.17 (±0.60)
Lay summary (*n* = 9)	2.56 (±3.09)	1.56 (±1.88)	−1 (±1.58)
Topic guide (*n* = 7)	1.29 (±1.80)	0.29 (±0.49)	−1 (±1.91)
Questionnaire (*n* = 2)	1 (±1.41)	1 (±1.41)	0 (0)
Assent form (*n* = 1)	2	1	−1
Protocol (*n* = 1)	0	0	0
Invitation letter (*n* = 1)	0	0	0

FAST-R, Feasibility and Support to Timely Recruitment for Research.

The final documents showed a significant reduction in the number of jargon words (before mean: 1.68 ± 8.23; after mean: 0.98 ± 5.44; *t*(98) = 2.37; *P* = 0.020; 95% CI 0.11–2.37), but documents became longer by 154 words (eight lines, or the equivalent of one paragraph) on average (before mean: 1199.4 ± 1116.6; after mean: 1353.3 ± 1336.4; *t*(98) = −4.64; *P* < 0.001; 95% CI −219.70 to −88.11). As patient-facing documents are important for consent, we separated them and found that participant information sheets became 12% longer after review, with an average increase of 275.41 words, and consent forms became 11% longer, but only by an average increase of 38 words.

Although there was no significant overall change in reading grade of the documents, 29.3% (*n* = 29) became easier to read (a negative grade), 12.1% (*n* = 12) did not change and 58.6% (*n* = 58) became more difficult to read. For participant information sheets, 20% (10 out of 49) improved, and for consent forms, 38% improved.

Most documents did not meet the recommended reading grade before FAST-R review (85%, *n* = 84), but six of these did meet the recommended reading grade after review (two consent forms, three participant information sheets and one lay summary). Of the 15 documents that initially met the recommended reading grade, three no longer met this criterion after review (two consent forms, one participant information sheet). So despite some improvements there were also detrimental changes.

### Did documents improve over time?

There were few changes over time in the documents submitted for review, except that there was an increase in the amount of jargon (*r* (*n* = 99) = 0.20, *P* = 0.046), and, following review, the number of suggested changes also increased (*r* (*n* = 99) = 0.31, *P* = 0.002), suggesting a lack of improvement over time.

### Do some documents produce more changes?

**Fig. 2:**
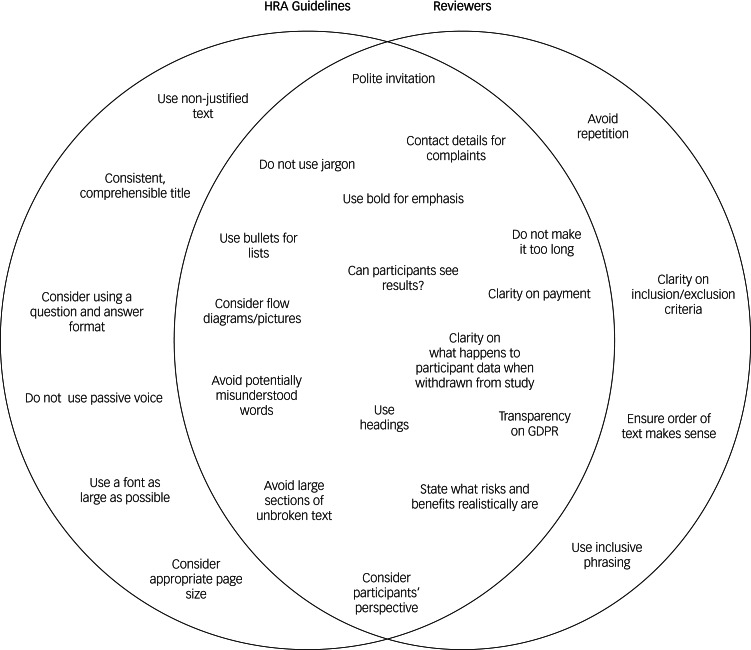
Comparison of FAST-R patient and carer guidance (right) with guidance from the Health Regulations Authority. FAST-R, Feasibility and Support to Timely Recruitment for Research; GDPR, General Data Protection Regulation; HRA, Health Regulations Authority.

Nearly all documents required changes (97 out of 99), with an average of 14 suggested changes to participant information sheets (Table 2), which were one of the most edited documents where researchers implemented more changes. Consent forms had few suggested changes (*n* = 5), with half implemented, and this may be because the Health Regulations Authority (HRA) provide direct guidance in the form of bullet points for researchers to use (see Fig. 2 for an overview of differences between HRA and FAST-R reviewer advice).

**Table 2:** The number of suggested changes made by FAST-R reviewers, by document type and the percentage of changes implemented by researchers

	Number of documents submitted (*n*)	Number of documents with suggested changes (*n*)	Number of suggested changes, mean (±s.d.)	Percentage of Suggested changes implemented, mean (±s.d.)
Overall	99	97	11.43 (±16.23)	62.25 (±31.72)
Document type				
Participant information sheet	49	48	13.92 (±18.86)	70.71 (±25.45)
Consent form	29	28	4.93 (±6.50)	50.01 (±41.20)
Lay summary	9	9	20 (±12)	67.70 (±13.66)
Topic guide	7	7	13.86 (±25.28)	45.43 (±32.96)
Questionnaire	2	2	10.50 (±10.61)	50 (±23.57)
Invitation letter	1	1	1	100
Protocol	1	1	2	50
Assent form	1	1	6	66.67

FAST-R, Feasibility and Support to Timely Recruitment for Research.

### What themes does FAST-R review produce for suggested changes?

With the inclusion of the feedback forms, we had 146 FAST-R documents. The analysis produced six categories: aspects valued by reviewers, issues around clarity, General Data Protection Regulation, language, study design and contact details, and presentation. A summary of the full framework with themes is shown in Table 3.

**Table 3:** Framework categories, and emerging themes with their subthemes

Framework category	Emerging themes	Subthemes	Occurrence,^a^ *n* (%)
Aspects valued by reviewer			144 (100%)
Clarity	Lack of information	Benefits of the study	13 (3.1%)
		Compensation for involvement	44 (10.7%)
		Practicalities	94 (22.8%)
		Purpose	53 (12.9%)
		Safety and mitigating risk	36 (8.7%)
		Side-effects and risks	11 (2.7%)
		Who is in the study team?	9 (2.2%)
	Inclusion and exclusion criteria		26 (6.3%)
	Terminology		82 (19.9%)
	Consistency (contradictions)		44 (10.7%)
General Data Protection Regulation	Consent		41 (29.5%)
	Transparency		51 (36.7%)
	Use of data (storage, length of time, withdrawal)		47 (33.8%)
Language	Grammar		30 (16%)
	Phrasing		129 (68.6%)
	Repetition		29 (15.4%)
Study design and contact details	Considerations about study design		94 (65%)
		Disseminating results of study	9 (6%)
		Recruitment	11 (8%)
	Contact details	Community or crisis services	7 (5%)
		Complaints	8 (5%)
		Research study team	16 (11%)
Presentation	Appearance		80 (42%)
	Order		41 (21%)
	Superfluous		71 (37%)

a. Occurrence refers to the number of documents coded to each theme, and percentage, relative to the framework category.

#### Aspects valued by reviewers

Reviewers noted when studies were ‘well designed and feasible’ and had real-life relevance (translational, e.g. ‘The reviewers feel that the study is worthwhile. Particularly as antipsychotics can lead to weight gain and other related physical problems’). Reviewers praised well-written documents so participants ‘could understand what they were being asked to do’ and contained substantial patient and public involvement. See quotes in Supplementary Table 2.

#### Clarity

Issues around a lack of clarity were common (see quotes in Supplementary Table 3), and several themes and subthemes emerged from this category.

##### Lack of information

Parts of documents lacked sufficient information, including how participants could benefit from the study (e.g. ‘the interaction from the education session and group walks’), compensation for involvement, study practicalities (e.g. ‘Reviewers were not sure what an eye tracker involved and felt that the procedure for this should be specified’), the study purpose (e.g. ‘How (the) research will ultimately benefit the population’), how participants would be kept safe (e.g. ‘what support is available for participants, should they become distressed’), side-effects and risks and how likely these are to occur (‘Reviewers hoped this risk could be quantified e.g. every one person in 100 who undergo this procedure’), and information on study team members.

##### Inclusion and exclusion criteria

Sometimes information on who could participate was lacking, which made it difficult for potential participants to know if they would be eligible. For example, if they were ‘allowed to be on medication’ or ‘involved in therapy’.

##### Terminology

Reviewers highlighted unclear terminology, such as jargon words and acronyms, and asked for further explanation on institutions or companies that were mentioned (‘The reviewers thought it would be good to spell out what NIHR stands for’). Issues with giving definitions for subjective issues were also highlighted (e.g. ‘recovery from anorexia’) and they suggested alternative words and phrases to explain jargon, as well as substituting negatively connotated words with more neutral terms (‘The reviewers felt that the terms “harmed” and “injured” could be interpreted negatively and suggested whether “affected” might offer an alternative?’).

##### Consistency (contradictions)

Reviewers highlighted contradictions and inconsistencies in the use of terminology and contradictory information on study procedures, e.g. its length, reimbursement, recruitment, people involved in the study and audio/video recordings.

#### General Data Protection Regulation

General Data Protection Regulation issues were central to many comments, and its themes are summarised in Supplementary Table 4. Reviewers particularly noted issues related to consent, such as the need for documents to be concise, as ‘this will make it more likely that participants will actually read it and be able to provide informed consent’, as well as transparency (e.g. ‘It is not mentioned that participants will be invited to attend a focus group or interview, or that a different protocol will be followed to gather information from service users and clinicians, and whether these will be audio recorded’) and how data will be used, storage methods, storage time and what will happen to data when participants withdraw from the study.

#### Language

Reviewers noted grammatical mistakes, misspelling and that ‘the tense should be consistent throughout’, as well as avoiding repetition (see Supplementary Table 5). Reviewers recommended using more inclusive phrasing (e.g. ‘In the gender section: the transgender option can be included’), as well as alternative phrasing of sentences in the documents that were difficult to read, unclear or could be offensive to the reader (e.g. ‘the term “diagnosis of schizophrenia” should be avoided as, from our experience, we find this can often cause offence, especially to those who do not believe they have this condition’).

#### Study design and contact details

Reviewers also commented on the study design and gave suggestions on contact details they felt were important to include (for a full breakdown see Supplementary Table 6). Reviewers queried ‘whether participants can ask to see a copy of the findings’, where participants would be recruited from and whether the amount of time allocated to different parts of the study process was realistic. Reviewers also provided suggestions, such as where to recruit participants and which contact details to include for community and crisis services, complaints departments and the research study team.

#### Presentation

Reviewers thought it was important that documents were aesthetically pleasing, and suggested using images, flowcharts, page numbers, and subheadings, as well as editing the text style and layout (e.g. formatting important sections in bold), breaking down paragraphs into bullet points and using checkboxes for demographic questions (see Supplementary Table 7). Researchers were recommended to ensure that the order of the text makes sense (‘We thought the section “Why are we doing the research” could be moved to below “What is the research about” as these sections complement each other’) and to avoid confusing or irrelevant information (‘We felt it would be more appropriate if the sentence starting “If you would like to take part … ” was removed to eliminate any confusion’).

### Which participant information sheets did reviewers prefer?

Most (six out of eight) reviewers preferred the post-FAST-R reviewed participant information sheets, and these documents were the ones that had more changes implemented (average 92.5%).

Comments by reviewers explaining their preference reflected those outlined above, with reviewers noting that the post-FAST-R reviewed documents were more transparent overall and contained the essential information, such as details on the study process, and the risks and benefits of the study. The language and layout of the documents were also frequently mentioned, with the post-FAST-R reviewed participant information sheets being more concise, in terms of ‘layout, wording and in anticipating some of the concerns that the potential participant might have’, as well as containing less jargon, using friendlier language (e.g. replacing the phrase ‘what will happen to me if I take part?’ with ‘what will happen if I take part?’) and being better structured overall. Those who preferred the pre-review information sheets provided similar reasons, stating the pre-reviewed versions were more concise and got to the ‘crucial information quicker’, as well as having a more coherent structure, being more succinct and using a preferable font.

### Tips for jargon busting

A list of jargon words/phrases along with alternatives were suggested by reviewers (see Table 4). Reviewers also highlighted other confusing terms, but were unable to provide alternatives (Table 5).

**Table 4:** Suggested alternative wordings by FAST-R reviewers

Terms used by researcher	Alternatives suggested by reviewers
Activities of daily life	Daily activities
Acquire	Collect
Altered	Changed
Application	App
Approximately	About
Assessment	(Short) test
Barrier	Hurdle
Clinical outcomes	Mental well-being
Compensated/reimbursed^a^	Paid
Confidential	Private
Deidentified^a^/pseudonymised	Anonymised
Detention	Compulsory admission/involuntary hospitalisation
Determinants	Risk factor/possible causes
Diet	What you normally eat
Disparities	Differences
Harmed/injured	Affected
Impact	Affect you
Impaired	Not working well
Individuals^a^	People
Informant	Carer or relative
Insufficiently responded	Treatment resistant
Intact	Working well
Key worker	Care-coordinator
Mental health disorder	Mental health problem
Novel	New
Opportunity	Chance
Participation^a^/participate	Taking part/take part
Provides to	Aims to
Psychopathology^a^	Mental health problem
Risk of relapse	Chance of becoming ill again
Shortly	Quickly
Smoking cessation	Stop smoking
Social environment	Community
Symptom questionnaire	Questionnaire about your symptoms
Treatment outcomes	How people get better
Undergo	Do
Voluntary	Up to you
Withdraw^a^	Stop and leave
You have a mental health disorder	You have been diagnosed with a mental health disorder/problem

FAST-R, Feasibility and Support to Timely Recruitment for Research.

a. Indicates terms that were frequently changed by reviewers.

**Table 5:** Words and phrases flagged up by reviewers as confusing or needing further explanation

Jargon words/phrases used by researchers	
Three-dimensions	NIHR BioResource
Adherence	Non-invasive
Anticipatory pleasure	Obligatory
MRC framework	Offence related trauma
Barriers	Open access journal
Biopsychosocial approach	Open dialogue (network meeting)
Coding your data	Outcome measure
Common mental health disorder	Parameters
Consent	Password protected
Consummatory pleasure	Peer-reviewed
*De novo* ethical approval	PERT chart
Disease state	Phlebotomist
Enablers	Prevalence
Encrypted	Principal investigator
Ethnography	Procedures
Feasibility study	Provision
Gaming	Quantitative study
Habitual competing responses	Quoracy
Informed consent quiz	Scope
Knowledge gap	Screening
Levels of inflammation	Sociodemographics
Mental function	Sustainability
Neurocognitive endophenotypes^a^	Upskilling

NIHR, National Institute for Health Research; MRC, Medical Research Council, PERT, XXX.

a. Relates to a specific medical study.

## Discussion

This was a mixed-methods audit to understand how patient and public involvement (through the FAST-R service) affects the accessibility of research documents, and highlights how to improve them. Although jargon words decreased, the word count increased after FAST-R review, indicating that patients and carers do not mind slightly longer documents if they are clear, and with no jargon. We show that there are many words and phrases that researchers may struggle to put into lay terms and we provide alternative. Researchers may also benefit from resources like the Patient Information Forum (https://pifonline.org.uk/), which provides a glossary of terms. The increased word count and decreased jargon also suggests that researchers did take the reviewers’ advice, and we found that the participant information sheets – a crucial part of informed consent – are among those edited most after review. We also found that all documents were edited after review. Despite these changes, there was no improvement in readability. We identified issues considered important, jargon words and phrases, and provide alternatives to improve accessibility. Importantly researchers did not seem to improve the documents, as over time, there was an increase in the amount of jargon in documents submitted for review. The increased jargon over time may be because HRA or research and development teams are increasingly mandating specific terms, which are inaccessible to lay people. When directly comparing pre-FAST-R and post-FAST-R documents, 75% (six out of eight) of participants preferred the post-FAST-R versions. Comments made by participants mirrored those of the initial qualitative analysis, indicating that researchers should strive to find a balance between providing enough essential information (e.g. on data protection, study process and risks and benefits) and avoid unnecessary information. When researchers implemented suggested changes, those documents were preferred, indicating that there is value in taking FAST-R reviewers’ comments seriously. Taken together, researchers can use this information to make research documents more accessible in the future.

We found more suggested and implemented changes in participant information sheets than consent forms, probably because consent forms may provide less flexibility with wording. Study sponsors may provide specific wording but the accessibility of these ‘stock’ phrases should be reconsidered. The HRA also publishes guidance for preparing participant information sheets and consent forms, and most of their guidance is echoed by the FAST-R reviewers in this study. These include recommendations for formatting, including headings, using bullet points, putting important aspects in bold, using friendly/inviting language and using diagrams and pictures. The HRA and reviewers also similarly emphasised the importance of not using jargon, and transparency on participant payment and use of data. The HRA, however, also suggest considerations that reviewers do not think are important, like page and font size. Reviewers brought up problems overlooked by the HRA, such as clear inclusion and exclusion criteria, inclusive phrasing (including the gender option of transgender), avoiding repetition and considering the order of the text. This reinforces the importance of involving patients in the creation of these documents, as they can identify ways to make them more accessible that are overlooked by other bodies.

The FDA recommend a reading level of grade eight or lower to facilitate adequate understanding of health information.^13^ We found only 15 documents in this study (15.2%) complied with this before FAST-R review. The National Adult Literacy Survey revealed that about a quarter of American adults could not read or understand written materials above a fifth-grade level,^27^ and in the UK, around 15%, or 5.1 million adults, have literacy levels at or below those expected of an 11 year old.^28^ In a readability assessment of 176 clinical research participant information leaflets and informed consent forms, several studies found few documents at this recommended readability grade.^1,29^ This highlights an urgent need to address accessibility issues, perhaps in the ethics guidance, so we ensure true informed consent.

Our FAST-R reviewers picked out similar issues that might prevent understanding by lay people, which replicates other studies,^2,30^ so this study emphasises the importance of these issues even for researchers who are not requesting patient involvement. It is important for everyone to consider the importance of true informed consent, rather than as an attempt to abide by enforced standards.

### Limitations

There are limitations with using the Flesch–Kincaid Grade Level measure to assess readability. The tool measures semantic and syntactic difficulties, without considering that documents containing jargon terms may use other means to improve readability, such as visual aids.^31^ Therefore, other measures may be more suitable to measure readability, such as the Clear Communication Index^32^ and the Suitability Assessment of Materials and future studies should aim to use these tools when assessing the impact of patient and public involvement.^33^

Additionally, the training the reviewers receive may mean that they have different standards for research documents, as well as what constitutes jargon, compared with lay patients. Therefore their views may not be representative of other patients who are less experienced in participating in research. Future studies could investigate whether there are differences in this regard between these participants and lay patients.

In conclusion, we have highlighted common mistakes made by researchers in documents submitted for patient review by FAST-R. These issues have not changed over time, and one issue that has been made worse is the use of jargon, so we provided a list of jargon words and phrases pointed out by FAST-R and ways to avoid them. This work not only shows the influence of involving patients, as all but two documents had changes made to them, but also shows the benefits of involvement at the earliest stage. Only through this involvement will we have true participant informed consent.

## Data Availability

The data that support the findings of this study are available from the corresponding author, S.J., upon reasonable request.

## Supplementary material

For supplementary material accompanying this paper visit http://dx.doi.org/10.1192/bjo.2021.1040. click here to view supplementary material
