# Supplementary material for: How to make study documents clear and relevant: the impact of patient involvement
Source: BJPsych Open. 2021 Nov 4;7(6):e202. doi: 10.1192/bjo.2021.1040 (PMC8596294; doi:10.1192/bjo.2021.1040)
Supplement: Supplementary file 1 [file S2056472421010401sup001.docx]

**Supplementary Material**

**Results**

**Part 1: Quantitative**

| Supplementary Table 1. Breakdown of document Types | |
| --- | --- |
| Participant Information Sheet | n = 49 |
| Consent Form | n = 29 |
| Lay Summary | n = 9 |
| Topic Guide | n = 7 |
| Protocol | n = 1 |
| Questionnaire | n = 2 |
| Assent Form | n = 1 |
| Invitation Letter | n = 1 |
| **Total** | **n = 99** |

**Part 2: Qualitative**

| **Supplementary Table 2: Examples of comments relating to “Aspects valued by reviewers”** | |
| --- | --- |
| **Framework category** | **Illustrative quotes** |
| Aspects valued by reviewer | *“generally, extremely supportive of this study and felt that it would be very beneficial to conduct a study to further understand the role of service users in research.”* |
|  | *“The reviewers thought that the PIS was well written, clear and covered all relevant information;”* |
|  | *“The reviewers feel that the study is worthwhile. Particularly as antipsychotics can lead to weight gain and other related physical problems, so it’s good to be addressing this.”* |
|  | *“very worthwhile project and well-written application. They were particularly pleased by the level of PPI planned for the project.”* |

| **Supplementary Table 3: Examples of comments relating to “Clarity”** | | | |
| --- | --- | --- | --- |
| **Framework Category** | **Theme** | **Subtheme** | **Illustrative quotes** |
| Clarity | Lack of information | Benefits of the study | *“the information sheet should clearly highlight possible benefits of participating for further reassurance”* |
|  |  | Compensation for involvement | *“Lastly the study does not mention how and if at all, it plans to reward the participants for their contribution? Is this something that has been considered?”* |
|  |  | Practicalities | *“How long is the study?”* |
|  |  | Purpose | *“It is important to mention the purpose of the research and potential benefits to the participants”* |
|  |  | Safety and mitigating risk | *“Due to the sensitive nature of the topic, reviewers felt more emphases needed to be placed on what support is available for participants, should they become distressed”* |
|  |  | Side effects and risks | *“Perhaps include a section about risks and benefits of taking part in the study”* |
|  |  | Who is in the study team | *“At the start could briefly mention who you are and where you work.”* |
|  | Inclusion and Exclusion Criteria |  | *“They also commented that there was no information about those who could and could not take part. For example, can people take part if they are on medication?”*    *“How would a potential participant know which medication might make you ineligible for the study?”* |
|  |  |  | Perhaps it would be useful to only have service users on the advisory group with a history of addiction and IPV? |
|  |  |  | The reviewers wondered whether previous experience of occupational therapy (or not) could affect participation in this study. |
|  | Terminology |  | *“However, the reviewer felt that particularly in the patient information sheet the term ‘offence related trauma’ should be explained further”* |
|  |  |  | *The reviewers felt that the terms “harmed” and “injured” could be interpreted negatively, and suggested whether “affected” might offer an alternative?* |
|  | Consistency (contradictions) |  | *“The title on the participant information sheet does not match what was stated in the protocol”* |

| **Supplementary Table 4: Examples of comments relating to “GDPR”** | | | |
| --- | --- | --- | --- |
| **Framework Category** | **Theme** | **Subtheme** | **Quote** |
| GDPR | Consent |  | *“The reviewers felt that the PIS is currently too long. If possible, aim for 4 pages. This will make it more likely that participants will actually read it and be able to provide informed consent.”* |
|  | Transparency |  | *“Make it clearer that sessions will be recorded.”* |
|  | Use of data (storage, length of time, withdrawal) |  | *“The reviewers felt a locked drawer was not safe enough for their identifiable data and that a locked filing cabinet would be more reassuring.”* |

| **Supplementary Table 5: Examples of comments relating to “Language”** | | | |
| --- | --- | --- | --- |
| **Framework Category** | **Theme** | **Subtheme** | **Quote** |
| Language | Grammar |  | *“Regarding the booklet, the reviewers suggested quite a few structural and grammatical changes to make it easier to read and understand.”* |
|  |  |  | *“The “Why have I been chosen?” section mentions what IAPT stands for, but says “increasing” rather than “improving”.”* |
|  | Phrasing |  | *“However we feel the term ‘diagnosis of schizophrenia’ should be avoided as, from our experience, we find this can often cause offence, especially to those who do not believe they have this condition.”* |
|  | Repetition |  | *We suggest that the sentence ‘We will give you a wallet sized summary of your top character strengths to keep’ is removed as it is repeated in the ‘what are the possible benefits’ section, where it fits better.* |

| **Supplementary Table 6: Examples of comments relating to “Study Design and Contact Details”** | | | |
| --- | --- | --- | --- |
| **Framework Category** | **Theme** | **Subtheme** | **Quote** |
| Study Design and Contact Details | Considerations about study design |  | *“The length of time spent in the clinic seems too long for children.”* |
|  |  |  | *“Could service users become involved in the training of staff in Phase 2 of the study and in running the qualitative focus groups?”* |
|  |  | Disseminating results of study | *“They also asked if findings would be available to participants.”* |
|  |  | Recruitment | *“They suggested having separate adverts for healthy participants and OCD patients.”* |
|  | Contact Details | Community or crisis services | *“The reviewers felt it may be helpful to include PALS contact details here.”* |
|  |  | Complaints | *“Under “Risks of taking part”, the reviewers felt that information was needed about who to contact if participants were unhappy with the study or wished to make a complaint.”* |
|  |  | Research study team | *“The name and contact details of the academic supervisor should be added to the information sheet.”* |

**Other comments coded to the theme “Considerations about study design” are summarised below:**

Reviewers commented on general issues relating to the design of the study, such as prior experiences affecting their performance in the study (I.e. if they have already carried out similar tasks if the past), preventing participants from dropping out (e.g. By considering when the treatment offered in the study will be most beneficial to participants and conducting focus groups prior to the study to assess acceptability) and reducing distress (e.g. by providing support resources). The comments made by reviewers suggested that there were concerns over involving children in the study (e.g. amount of time they will be involved for, reimbursements given to children), asking too much of participants (tasks may be too extensive and exhausting), unrealistic goals set by the research team (e.g. budget, sample size, amount of time it will take to complete a part of the study) and commented on technological issues that may arise. The reviewers wondered whether service users could become involved in parts of the study and whether carers could be involved and how. Reviewers also made suggestions for considerations when writing up the study, relating to confounding variables, issues with small sample sizes and suggested considering reducing the number of variables in the research.

| **Supplementary Table 7: Examples of comments relating to “Presentation”.** | | | |
| --- | --- | --- | --- |
| **Framework Category** | **Theme** | **Subtheme** | **Quote** |
| Presentation | Appearance |  | *“They suggested breaking down larger paragraphs into smaller ones and use of bullet points to make it more reader friendly.”* |
|  | Order |  | *“The reviewers felt this summary paragraph may be helpful at the top of the page to summarise the study to the participant straight away.”* |
|  | Superfluous |  | *“There is a lot of information about mental imagery. Is it necessary to describe it in so much detail?”* |
